# The Interplay of Nutriepigenomics, Personalized Nutrition and Clinical Practice in Managing Food Allergy

**DOI:** 10.3390/life11111275

**Published:** 2021-11-22

**Authors:** Adli Ali, Nur Hana Hamzaid, Noor Akmal Shareela Ismail

**Affiliations:** 1 Department of Pediatrics, Faculty of Medicine, Universiti Kebangsaan Malaysia, Kuala Lumpur 56000, Malaysia; adli.ali@ppukm.ukm.edu.my; 2 Dietetic Program & Centre for Rehabilitation and Special Needs Studies (iCaRehab), Faculty of Health Sciences, Universiti Kebangsaan Malaysia, Kuala Lumpur 50300, Malaysia; hanahamzaid@ukm.edu.my; 3 Department of Biochemistry, Faculty of Medicine, Universiti Kebangsaan Malaysia, Kuala Lumpur 56000, Malaysia

**Keywords:** nutriepigenomics, personalized nutrition, avoidance diet, food allergy

## Abstract

Food allergy in children has been a common issue due to the challenges of prescribing personalized nutrition with a lack of nutriepigenomics data. This has indeed further influenced clinical practice for appropriate management. While allergen avoidance is still the main principle in food allergy management, we require more information to advance the science behind nutrition, genes, and the immune system. Many researchers have highlighted the importance of personalized nutrition but there is a lack of data on how the decision is made. Thus, this review highlights the relationship among these key players in identifying the solution to the clinical management of food allergy with current nutriepigenomics data. The discussion integrates various inputs, including clinical assessments, biomarkers, and epigenetic information pertaining to food allergy, to curate a holistic and personalized approach to food allergy management in particular.

## 1. Introduction

Food allergy has been a problem affecting humankind since more than 4500 years ago. The earliest evidence of food allergy was first appreciated in the Chinese literature (~2750–2650 BC), through a practice of “Shi Jin-Jing”, in which individuals suffering from certain skin lesions and pregnant women were advised to avoid certain foods particularly those containing shrimp and meats [1]. Later, Hippocrates (460–377 BC) proposed the role of IgE antibodies in food allergy, describing them as “hostile humors’’ in individuals with allergic reactions upon ingestion of cheese [2]. Although the presence of food allergy had been recognized well before, it was Praustnizt and Kutsner exactly a century ago, who explained scientifically the immunological basis of our current understanding of food allergy reactions [3]. In their landmark experiment, Praustnizt, using Kutsner’s fish-sensitized serum, demonstrated a factor in Kutsner’s serum (which was subsequently identified as IgE) as the main factor driving the pathology in the development of specific food allergy [3].

Since then, food allergy has emerged as a growing concern worldwide and is even considered as the ‘second wave of the allergy epidemic’ [4]. Food allergy incidents are increasing in recent years, with a prevalence in children ranging from 6.53% to 11.0% [5,6,7,8,9,10]. The prevalence of food allergy in Asia is widely variable, but recent studies show the prevalence is comparable to that in western countries, ranging from 3.4–6.4% and 5.3% among children in Taiwan and Korea, respectively [11,12]. Despite the increasing trend of food allergy globally, the disease tends to be ignored by most stakeholders and health-funders, especially among developing countries. The management of a child with food allergy on average was documented to cost USD 4184 per year, thus is a significant economic impact on the patient’s family [13]. More disturbing is the fact that food allergy has been reported to have a detrimental effect on the quality of life of the patients and caretakers, making food allergy one of the debilitating diseases in the current health era [14,15].

## 2. Disease Management and Current Clinical Challenges

Children with a food allergy may manifest multiple types of allergic reactions. The most common clinical manifestation of food allergy among children less than 2 years old was found to be skin manifestation (65.7%), presenting either as acute urticaria or chronically as chronic dermatitis [16]. Gastrointestinal symptoms such as vomiting and diarrhea is also a common manifestation among younger children with food allergy [16]. In children 2 to 10 years old, respiratory tract symptoms, such as rhinoconjunctivitis (74.5%) and asthma (36.9%) were found to be more prevalent [16]. These combinations of a potentially acute respiratory manifestation and severe anaphylaxis emergency presentation, in addition to the chronically disturbing skin and gastrointestinal symptoms, make food allergy a challenging disease to manage for both patients and the parents.

With food being the main culprit in this disease, the differences in dietary habits in different races and regions of the world have influenced the list of common allergens within the particular region or race [17]. For example, the incidence of peanut allergy is not highly prevalent in the Southeast Asia population compared to Western countries, while the incidence of allergy towards cashew nuts was observed to be comparatively higher among children from the Southeast Asia region [18,19,20]. Nonetheless, the main allergens remain largely true to most parts of the world, with eggs, milk, fish, and shellfish being the top four main food allergens, while peanuts, tree nuts, soybeans and wheat are the next four items, thus completing “the big eight” common food allergens [21,22,23].

Early and accurate diagnosis is important in the management of food allergy among children. Nonetheless, the complexity of the disease complicates the diagnosis and the intervention strategies. Oral food challenges, by far, remain the gold standard in investigating children with suspected food allergy [24,25,26]. Although double-blinded placebo-controlled oral food challenge is theoretically the best approach, open and single-blinded oral food challenge among children is widely acceptable and less cost- and time-consuming [25,27,28]. However, this approach is limited in its application for preventive measures, pre-requisitely requiring an established center and well-trained experts. Allergen-specific skin prick tests or measurements of antigen-specific serum IgE level are cheaper, less labor-intensive and more practical investigations that can be done in a smaller and clinic setting [24,26,29,30]. Nonetheless, both tests might be restricted with the availability of locally important allergens to the region, and precautions need to be taken in the interpretation of the result, especially with the lack of correlating clinical symptoms. The cross-reactivity phenomenon is another crucial aspect that needs to be carefully considered in the management of children with food allergy, as misinterpretation might result in an unnecessary elimination of diets [24,31]. Quantification of specific IgE to individual allergenic components provides a solution to distinguish genuine versus cross-reactive sensitization, which is useful in guiding personalized nutritional management [32,33]. Yet, the accessibility is vastly restricted in lower-resource settings, and as described in other investigations, its usefulness is also limited by the breadth of individual components available for testing [34,35,36]. A huge downfall in the current approach is that it is only focusing on the management of patients with established development of food allergy. What is hugely lacking and should be the future approach, is predicting who will be developing and significantly manifesting the clinical symptoms of food allergy.

## 3. Dual-Allergen Exposure Hypothesis and Shifting of Nutritional Intervention

The conventional standard strategy in the management of food allergy used to be dependent on removing causative food from the children’s diet [37,38]. However, this older approach was not just proven to be challenging but there is building evidence suggesting a pure elimination diet might be futile in the prevention of food allergy development [39,40,41,42]. With the rise of food allergy among children in recent years, genetic predisposition alone is not adequate to explain this, and changes in environmental factors are suggested to be a main contributing factor to this current phenomenon. Several factors have been proposed, with the dual-allergen exposure hypothesis being implicated as the present contemporary notion [43,44].

The dual-allergen exposure hypothesis is based on the principle of earlier pathogenic sensitization towards a certain food allergen, occurring through a breakdown of the skin barrier, resulting in an allergic reaction upon subsequent oral ingestion of food [45,46]. This theory is strengthened by the common clinical observation as well as explaining the manifestation of eczema as the main risk factor in a child for developing a food allergy [47]. Two studies, namely the LEAP and EAT, further supported this notion of dual-allergen exposure hypothesis and challenged the previous dogma of pure diet elimination role in the management of food allergy [48,49]. It was shown in these two landmark studies that earlier and routine introduction of allergenic food in high-risk infants as early as three months of age reduced the risk of developing food allergy [48,49]. Based on this, the National Institute of Allergy and Infectious Diseases (NIAID), the American Academy of Pediatrics, and the American Academy of Allergy Asthma and Immunology all recommended the early introduction of allergenic food in high-risk infants for the prevention of food allergy [42,50].

Despite the documented evidence and the current recommendations, the challenge in implementing the shift in nutritional interventions are multifaceted. This includes (i) introduction of diverse types of food including a selection of the intended allergenic food, (ii) adherence and sustained feeding to reduce food allergy development risk, and (iii) identifying infants to be considered at risk of developing food allergy later in life [51]. Guidance to correctly determine which babies are at risk of food allergy, thus benefiting from this nutritional intervention, will be helpful in convincing parents and ensuring compliance to the intended nutritional intervention. Additionally, precisely knowing the types of allergenic foods to be emphasized during this early dietary introduction allows for a structured dietary program to be planned between the parents, physician and the dietitian. Moreover, the inception of a food allergy towards a different food allergen is variable, thus knowing exactly which and when the timing of this pathogenesis happens permits understanding when a particular diet tolerance induction program should be initiated [52].

Genetics, although not solely, plays an integral part in the development of pediatric food allergy. Genetic role in the development of food allergy is clearly documented through observations of higher concordance in monozygotic twins compared with dizygotic twins, albeit less than 100% thus emphasizing the influence of other factors such as the environment [53,54]. The interplay of the human genome and nutrition through exploration of the role of nutriepigenomics may deepen our current understanding of the pathophysiology of food allergy among children.

## 4. Nutriepigenomics

Epigenetics is a process of how the changes in nutrition intake could influence food allergy through the biochemical changes at the molecular level of our body. This alters our gene expression through the process of opening and closing histone proteins, to further opening the chromatin and allowing DNA to be more accessible. The advancement of research in both nutrition and genomics has paved the integration of these words. With the development of omics technologies, researchers have hypothesized the involvement of epigenetics in the manifestation of food allergy, thus the newly coined term has emerged, namely nutriepigenomics. This term is closely related to nutrigenomics which refers to how DNA sequence variation is responding to nutrients whilst nutriepigenomics is focusing on the role of nutrients in overexpressing or silencing a specific gene [55]. Extensive investigations on nutriepigenomics were based on acute and repeated exposure to the environment, specifically a combination of nutrients. Since the evolution throughout human history, different types of nutrition have evolved and further influenced gene expression for more adaptive phenotypes to survive different environmental challenges [56]. These adaptations have been a crucial driving factor to push for human growth and development and might interfere with the immune response. Therefore, with the new role of nutriepigenomics in food allergy along with the progression of DNA technologies [57], it is possible to explore a genome-wide study in a specific population to look for various and possible DNA sequence variants before proposing better management.

## 5. The Role of Nutriepigenomics in Food Allergy

The development of food allergy requires a certain interaction through prolonged exposure to the nutrient intake since in utero. Numerous twin studies and/or sibling studies revealed there is an increased rate of sensitization which does not equate to reactivity to the allergens. Most studies that are linked to food allergy have highlighted the expression of genes that produce signaling proteins that in turn activate the downstream stimulation of the T cell helper (Th2) phenotype. Th2 is one of the key players that mediate the recruitment of IgE to further produce B-cells, mast cells and eosinophils in activating allergic reactions. Various epigenetic changes affecting DNA methylation at the promoter regions and increasing/decreasing the rate of histone acetylation were seen in different genes encoding signaling proteins involved in immune responses which subsequently regulate downstream protein production in allergic inflammation. For example, epigenetic changes in the *FOXP3* gene influence regulatory T cells (Treg) function, which are responsible for suppressing immune responses [58], whilst epigenetic changes in the *PGM3* gene affect glycosylation of Treg, thus changing the immunoregulation responses [59].

## 6. DNA Methylation Affects Gene Expression in the Presence of Food Allergens

DNA methylation is a process of an additional methyl group onto cytosine and is frequently found in the cluster of CG repetitions. It is typically located in the gene regulatory element at either promoters or enhancers which impacts its transcriptional activities [60,61,62,63,64]. When a promoter region is being undermethylated, this allows a series of genes to be transcribed and further translated whilst the hypermethylation is linked with the switching off of the gene expression. The increased rate of DNA methylation can be influenced by the nutrients and is suggested to be one of the processes to initiate allergic reactions, especially in food allergy [65]. This process was seen to activate the gene expression of cytokines which furthers the process of cell differentiation of T cell helper (Th) into Th1, Th2, Th17, or Treg phenotypes [66,67,68]. 

The latest next-generation bisulfite sequencing allows greater coverage of each CpG site at promoter/enhancer regions at genes of interest. This technique has evidently evaluated DNA methylation levels on 70 immune-related genes to address the association between methylation at the CpG sites of these genes to the response of peanut allergen [69]. In peanut allergy, 12 genes were hypermethylated, of which 7 of those were potentially novel to food allergy, 3 genes were associated with Th1/Th2 responses, and 2 genes were associated with innate immunity [69]. Hypermethylation at CpG sites also occurred at both *HLA-DQB1* and *HLA-DRB1* genes, which are also involved in food allergy, and such effect can be seen through the presence of a single nucleotide polymorphism [70]. 

Genetic variants in filaggrin (*FLG*) have been suggested to be associated with the increased risk of food allergy, with an association with peanut allergy specifically [71]. *FOXP3* demethylation was associated with the activation of Treg cells linked with peanut allergy [72,73]. Multi-omics approaches [57] have elucidated mechanistic pathways on how food allergies are manifested, including the role of T cells and B-cells in peanut allergies among affected infants [74,75]. Additional epigenetic regulation of *C11orf30*/*EMSY*, *SKAP1*, and *CTNNA3* is also associated with the development of peanut allergy [76]. This indicates a better diagnostic biomarker in comparison to serum IgE. However, there are a variety of responses between peanut allergy and no allergy samples that could indicate the interaction of these genes with the environmental factors [69].

## 7. Histone Acetylation Allows Gene Accessibility to Promote Allergy Reaction

Histone acetylation is one of the major chromatin epigenetic modifications that have been shown to allow access to increase the rate of gene expression as a reaction to different types of food allergy. When a histone is acetylated at the N-terminal tail, it allows gene transcription through the opening of the histone. Increased rate of acetylation at the subunit H3 and H4 of histones leads to the opening of the chromatin which eventually leads to better accessibility of promoters for transcription for higher gene expression [77]. However, a stimulus from a certain nutrition intake will deacetylate the histone and reduce the rate of transcription. Diet including fish and/or olive oil among pregnant mothers could affect the histone acetylation in the placentas, thus affecting the newborn specifically at H3 subunit where *FOXP3*, *IL10RA*, and *IL7R* genes are located [78]. Additionally, fish consumption among mothers is significantly correlated with increased H4 acetylation at the *CD14* gene in the placentas [78].

The observed histone acetylation changes are also seen in cow’s milk allergy. In comparison between raw milk and processed milk, histone acetylation of *Th1-*, *Th2-*, and regulatory T cell-related genes of splenocyte-derived CD4+ T cells was found to be higher in raw milk than in processed milk exposure [79]. After first exposure and allergic reaction and resolved, histone acetylation of *Th2* genes was found lower in the raw milk when compared to processed milk [79]. In another study looking at the effect of cow’s milk allergy, a reduced percentage of regulatory T (Treg) and T helper 17 (Th17) cells were prevalent, in parallel to decreased levels of H3 and/or H4 histone acetylation at *Treg* and *Th17* loci [80]. This indicates that activating T cell-related genes can affect the tolerance to milk, and additionally the exposure to raw milk exhibits an allergy-protective effect through the epigenetic modifications of T cells.

Allergic reactions can also be mediated by the IgE-mediated mast cell activation as a response to food antigens. This is suggested to be influenced by histone acetylation induced by the dietary substances which later activate the mast cells, especially in acute food allergy reactions that often correlate with severe gastrointestinal issues [81]. This effect is suggested to involve the cell signaling within the mast cells and is related to the activation of Th2 cells [80,82]. In the presence of whey-specific IgE, histone acetylation level is increased to further augment *STAT6* gene expression [80]. IgE is also related to varieties of IgE binding proteins that react well to parvalbumin in a fish allergen [83,84] and tropomyosin as crustacean allergen [85]; however, their molecular mechanisms and interaction have not been yet elucidated on how methylation and acetylation could lead to the rising of the allergies [86,87]. However, the allergic reaction to certain shellfish may be related to the modulation of *HLA* genes [88]. 

The *Treg* modulation towards a Th2-cell-like lineage has been shown to impair oral tolerance and could possibly promote the incidence of food allergy. Th2-type immune response was observed in elevated egg-specific IgE and IgG1 antibodies level, and concomitant increase production of FOXP3 and Treg [89] in egg allergen and IL4, IL-5 proteins [90] in soybean allergen, which suggested the epigenetic changes at the highlighted genes. Additional activation of IL-4R signaling also diminished the production of STAT6-dependent and functional mucosal allergen-specific Treg cells correlated with the Treg cells recruitment by Th2-cell-like phenotype [91]. 

## 8. The Role of Environmental Modulators of Nutriepigenomics

**Figure 1:**
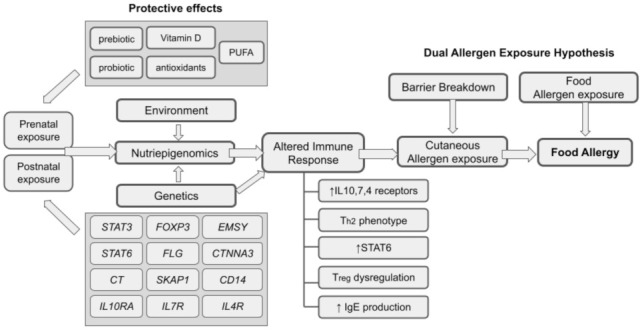
An overall relationship of nutriepigenomics and food allergy.

Genetic predisposition is unique from one individual to another and how the set of the genome responds with acute and prolonged environmental exposures determine the food allergy etiology. Therefore, it is crucial for all medical practitioners, scientists, and dietitians to scrutinize the data from nutriepigenomics to see the relationship between the gene–environment interactions [92]. Environmental factors can affect the rate of DNA methylation to further heighten the chances of getting a food allergy. DNA methylation and acetylation of histones are indeed the important mediators of gene–environment interactions in food allergy and are becoming a major key to understanding the mechanisms underlying allergic development. Prenatal and postnatal exposures involving interplays of multiple genetic and environmental factors predispose an individual toward food allergy (Figure 1). Specifically, the exposures of these environmental factors in tandem to the related immunity-genes, result in epigenetic changes that alter immune responses, which subsequently predispose them towards the development of food allergy. The ‘dual-allergen exposure’ hypothesis emphasizes the role of earlier sensitization of the predisposed individual towards a particular antigen by cutaneous allergen exposure via breakdown of the skin barrier in the form of chronic dermatitis lesion [47]. The chronic cutaneous allergen exposure in combination with the altered immune response in at-risk individuals set the stage for the allergic responses, upon oral consumption of the particular allergen.

There are a variety of protective effects of nutrients that have been shown to influence the epigenetic markers and reduce the severity of food allergy. A good diet should be advised among pregnant mothers that consists of antioxidants, vitamin D, folates, and polyunsaturated fatty acids, whilst exposure to external smoke changes the epigenetics and induces allergies, thus should be avoided. Long exposure to secondhand smoke whether in utero or after birth increases the chances to develop several allergic diseases including food allergy [93]. This may further exacerbate to become another allergic reaction including asthma and atopic dermatitis which is plausible in relation to the persistent “Th2 state” [94]. The suggested modulators have been proposed to change nutriepigenomics, including the fetal programming, in either excess or deficient amounts. Antioxidants are known for their ability to combat cell damage through reactive oxygen species, and this is evident in their effects on the histone deacetylase genes [95]. Vitamin D is also known to modulate chromatin in several immune cells [96]. The deficiency of this vitamin is significantly correlated with multiple food allergies, especially in peanuts [97].

Prebiotic and probiotic supplementation can directly affect the gut, especially the microbiota and immune system [98,99], which starts before birth under an intrauterine environment [100]. Studies have highlighted the importance of this supplementation and traced back the effects on the gut microbiota among pregnant mothers and transferred the effect to the offspring through an intrauterine environment [101,102,103]. Supplementation with probiotics among pregnant women has influenced the demethylation of DNA in gene promoters [104,105,106]. The introduction of many strains of *Lactobacillus* and *Bifidobacterium* further modulate immune response especially in Th1/Th2 balance [100,107] and regulate the immune system especially in T (Treg) cell development [108,109]. The epigenetic modulation of *Th1/Th2* gene expression in an in vitro model highlighted the role of NF-kB and different interleukins that can be influenced by the presence of probiotics, through epigenetic biomarkers [110]. Probiotics also were shown to decrease inflammation with a high production of butyrate in the gut [111]. Probiotics also improve the symptoms of allergic diseases by elevating IL-4, IL-10/IFN-γ, and Treg/TGF-β, whilst serum eosinophil levels and the expression of metalloproteinase-9 were seen to be reduced significantly [109]. Additionally, prebiotics supplementation (fructo-oligosaccharides) can improve gut defense and immune response [112,113] whilst galactooligosaccharides and inulin mixture prevent food allergy [102,114] by affecting the Th1/Th2/Th17/Treg balance [115].

## 9. Personalized Nutrition in the Management of Food Allergy

Personalized nutrition has gained a spotlight in the last ten years as a proposed management for food allergy. Earlier personalized nutrition was based on food avoidance from the data gathered from the Food Frequency Questionnaire (FFQ), especially the frequency of food intake that triggers allergic reaction. The current movement to advocate general nutrition advice is based on various factors, including the anthropometric data, meal content, activity tracking and dietary intake data. Only recently, a similar approach has been conducted for food allergy, with additional clinical and biochemical parameters, microbiome and genetics for each individual [116,117,118].

The growing evidence of nutriepigenomics in response to food allergy has led to understanding how precisely food allergy should be managed. The elucidation of molecular mechanisms based on several omics technologies [55] should assist in the translation of genetic information into nutritional recommendations. Therefore, the identification of genetic variants and epigenetics biomarkers should steer the direction towards personalized nutrition based on genetic make-up [119,120]. We now propose how the current evidence on nutriepigenomics can be integrated into the current clinical management towards personalizing dietary interventions.

The current practice in dietary management is based on the results obtained from several types of diagnostic testing, such as history taking for the investigations for food allergy in identifying IgE-mediated food allergy that further can be confirmed by food challenges or elimination diets [121]. The results are interpreted as types of food to be removed from the diet, in which dietitians later analyze the adequacy of nutrients based on the standard requirements of each individual to suggest any food alternatives to be prescribed to the individual, to compensate for the deficiency from any of the food avoided to ensure healthy growth of individuals, particularly children and patients at risk of malnutrition. This approach, however, took place when signs of anaphylactic shock had occurred.

It is understood that the key management for the long-term treatment of patients with a food allergy is food avoidance [122]. The biggest concern of this approach is the high risk of malnutrition and further exacerbating feeding difficulties among children [123], especially in picky eaters. It is also known that some food can be introduced gradually to allow the body to build a tolerance to the food later in life. This point is very hopeful in providing a more wholesome and balanced diet that will lead to better life quality. With the new data obtained from the epigenetic testing, one individual’s potential allergy, namely from food, can be identified early with more precise information to guide and personalize dietary management, mainly among children with a strong family history of food allergy [117]. Perhaps not just food, but more certain substances that may trigger the allergic reaction can be identified. If this can be understood, more strategies can be developed, as for example, there are certain cooking techniques that can be performed to denature the trigger elements so that the individuals are able to enjoy the food and not totally eliminate it from the diet.

Examples of food allergens such as peanut, tree nut (cashew, Brazil nut, pistachio), crustaceans, and mollusks, are crucial to identify earlier in life and avoid them all at once. However, the current practice of eliminating them totally from the diet may not be so relevant when the nutriepigenomics approach is explored further. The foods can be introduced gradually in several phases, together with additional cooking techniques that can be taken by the families [124,125]. This will later lead to a more structured food allergy management, as for example, categorizing food allergy into several types of dietary intervention techniques from a full dietary elimination to foods that can be introduced gradually.

**Figure 2:**
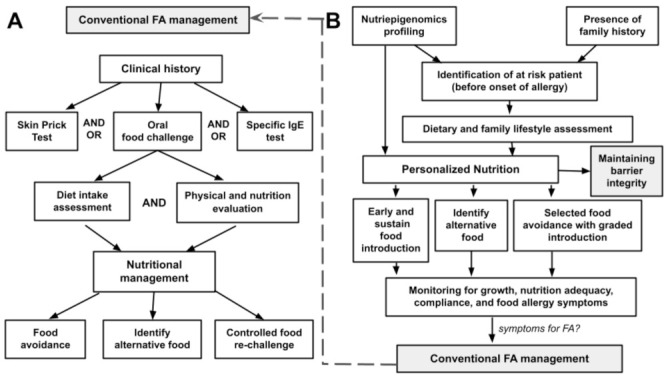
(**A**) A conventional food allergy (FA) management and (**B**) the role of nutriepigenomics in personalized nutrition.

Figure 2 depicts both conventional and newly proposed food allergy management. Conventional food allergy (FA) management focuses on patients who are clinically suspected or confirmed with food allergy (Figure 2A). Detailed clinical history is the central part of this management approach with ‘exposure-to-symptoms’ providing the primary information on the potential allergens. Subsequent investigations either in the form of skin prick test, specific serum IgE measurement and/or controlled oral food challenge are utilized to correlate with the clinical history obtained, as well as exploration of other unidentified allergens. Dietary assessment and physical evaluation of the individual are carefully considered in the planning of the intended nutritional intervention. This nutritional management conventionally focuses on providing a list of foods to be avoided, identifying alternative foods or specific nutrients that possibly compromised the elimination diet planned, and eventually in part, may include re-challenge of some category of food later. 

The proposed personalized nutrition intervention in food allergy shifts the focus towards prevention and early allergenic food introduction (Figure 2B). The central part of this proposed strategy is based on the identification of at-risk patients before the onset of food allergy, through the integrated knowledge obtained by nutriepigenomics profiling of the individuals in addition to other known or possible risk factors such as the presence of family history. Upon identification of the potential individuals, lifestyle and dietary assessment will be evaluated, and the pieces of information from the nutriepigenomics profiling will be utilized to structure a personalized nutritional intervention. The approach of this nutritional intervention will include (i) determination of food types and the timing of the early/regular food introduction, (ii) providing appropriate food alternatives without compromising nutritional value and local lifestyle, and (iii) identifying food groups that need to be initially eliminated before introducing in a timely, gradual, and controlled manner. Monitoring of growth and compliance of this personalized nutritional intervention with surveillance of allergy symptoms is part of the follow-up intervention in this nutritional management plan. In any case, with allergies developed towards a particular food allergen, the previously described approach in Figure 2A is implemented.

## 10. Conclusions

Nutriepigenomics data should be integrated into the current best-practice guidelines for the proper management of food allergy. Health professionals should be updated with the current molecular findings and these findings should be used as a compulsory tool in assisting the best personalized dietary recommendation. Thus, this review has collated numerous pieces of evidence to corroborate the interlink and interplay of nutriepigenomics and food allergy management. However, the potential link between epigenetics and food allergy needs to be elucidated with the involvement of various omics platforms so we can further personalize management for each patient based on their genetic profile. The monitoring symptoms of the food allergy for each individual should be continuous, and it is best to start at an early age. Indeed, the modification and identification of the mechanistic pathways have unlocked a bigger avenue towards an emerging new food allergy prevention through a personalized approach.

## Data Availability

Not applicable.
